# Supplementary material for: Analysis of the Psychosocial Impact of the COVID-19 Pandemic on the Nursing Staff of the Intensive Care Units (ICU) in Spain
Source: Healthcare (Basel). 2022 Apr 25;10(5):796. doi: 10.3390/healthcare10050796 (PMC9141599; doi:10.3390/healthcare10050796)
Supplement: Supplementary file 1 [file healthcare-10-00796-s001.zip › healthcare-1668137-supplementary.pdf]

Supplementary Material

Annex I—Questionnaire

**Socio-demographic data**	
Age	Short answer text: Insert age in number.
Sex	○ Man<br>○ Woman<br>○ I prefer not to say
Please indicate in which hospital you worked in the ICU during the pandemic (you can select more than one)	Short answer text
Please indicate in which wave of the pandemic you have worked in the Intensive Care Unit **(**you can select more than one)	○ First wave<br>○ Second wave<br>○ Third-wave<br>○ All
Employment relationship	○ Temporary<br>○ Fixed<br>○ Interim
**Perceptions of working conditions during the pandemic**	
What do you consider your level of exposure has been within the unit? *	○ No or very low grade<br>○ Low grade<br>○ Medium grade<br>○ High grade<br>○ Very high grade
Do you believe that you have been provided with adequate working	○ Yes

conditions to cope with the situation since the beginning of the pandemic? *	○ No
If not, do you think it has subsequently changed?	○ Yes<br>○ No
Have you been provided with all the assistance needed, both material and personnel? *	○ Yes<br>○ No
Do you think our healthcare system was prepared for this situation? *	○ Yes<br>○ No
Why do you think so?	Long answer text
**Perception of mental health status**	
Have you felt more worried (either about your or your family's health, work, or anything related to the pandemic)? *	○ No, I have not been worried.<br>○ Just as worried as ever.<br>○ Somewhat more worried than usual.<br>○ Much more worried.
Have you felt more tired? *	○ No, I have never felt tired.<br>○ Just as tired as ever.<br>○ Slightly more tired than usual.<br>○ Much more tired than usual.
Have you had sleep problems? *	○ No, I have never had any sleep problems.<br>○ The same as always.<br>○ Somewhat more than usual.<br>○ Much more than usual.
Have you ever felt fear? To what degree? *Understand fear as the fear you feel when considering something harmful, such as returning home with	○ No, I have never been afraid.<br>○ Yes, to the same extent as always.<br>○ Yes, a little more than usual.

the possibility of infecting other people, fear of the unknown... *	○ Yes, much more than usual.
Indicate the degree to which you have felt anxiety. *Anxiety is the mental state of anguish and alertness that occurs in the face of a threat and is characterised by great restlessness, excitement and insecurity. *	○ I have never felt anxiety.<br>○ The same as always.<br>○ Somewhat more than usual.<br>○ Much more than usual.
Do you consider that you have suffered from psychological stress? To what degree? *Psychological stress is defined as negative or unpleasant emotional reactions to a situation of excessive demand. These reactions are often anger, anxiety and depression. *	○ No, I have never felt it.<br>○ I felt the same as always.<br>○ Somewhat more than usual.<br>○ Much more than usual.
Have you ever felt down, depressed or hopeless? *	○ Yes.<br>○ No.
Have you had times when you felt you had lost your vocation or even considered quitting your job? *	○ Yes.<br>○ No.
Could you tell what feeling or psychological state has characterised each pandemic wave for you?	Long answer text.
Are you aware of the counselling services offered in your hospital to professionals? *	○ Yes.<br>○ No.
Have you had to seek psychological or psychiatric help? *	○ Yes.<br>○ No.<br>○ No, but I think I need it.
Has the use of psychotropic medicines increased? *	○ No, I consume the same as always.<br>○ Yes.<br>○ I have never used it.

**Life events**	
Have you experienced any other traumatic events related to the pandemic (such as your own or a family member's infection, someone close to you being hospitalised or dying from COVID-19...)? *	○ Yes.<br>○ No.
Has the COVID-19 virus infected you? *	○ Yes.<br>○ No.
**Social relations**	
Have you experienced fear of infecting your immediate family/social environment? *	○ Yes.<br>○ No.
Do you have people at risk of COVID-19 in your immediate environment? *	○ Yes.<br>○ No.
Are you a risk person?	○ Yes.<br>○ No.
Have you changed your behaviour towards those close to you, or even felt compelled to take isolation measures? *	○ Yes.<br>○ No.
Do you feel that your relationships have been affected by the situation you have faced? *	○ Yes.<br>○ No.
**Labour relations**	
How has the relationship with your colleagues been? *	○ Better, we worked as a team, and I felt supported.<br>○ Same as ever, no better, no worse.<br>○ Worse.
Do you think this has harmed your work performance and, therefore, the quality of patient care? *	○ Yes.<br>○ No.

Have you experienced difficulties in focusing on your work? *	○ Yes.<br>○ No.
